# Changes of adenosine deaminase activity in serum and saliva around parturition in sows with and without postpartum dysgalactia syndrome

**DOI:** 10.1186/s12917-021-03067-6

**Published:** 2021-11-18

**Authors:** Marianne Kaiser, Jan Dahl, Stine Jacobsen, Magdalena Jacobson, Pia Haubro Andersen, Poul Bækbo, Damián Escribano, José Joaquín Cerón, Fernando Tecles

**Affiliations:** 1 Department of Animal Science, Aarhus University, Blichers Allé 20, 8830 Tjele, Denmark; 2 Danish Agriculture and Food Council, Axelborg, Axeltorv 3, 1709 Copenhagen V, Denmark; 3 Department of Veterinary Clinical Sciences, Faculty of Health and Medical Sciences, University of Copenhagen, Agrovej 8, 2630 Taastrup, Denmark; 4 Faculty of Veterinary Medicine and Animal Science, Department of Clinical Sciences, Swedish University of Agricultural Sciences, P.O. Box 7054, SE-750 07 Uppsala, Sweden; 5 SEGES, Danish Pig Research Centre, Agro Food Park 15, 8200 Aarhus N, Denmark; 6 Department of Animal Medicine and Surgery, Regional “Campus of Excellence Mare Nostrum”, University of Murcia, 30100 Espinardo, Murcia, Spain

**Keywords:** ADA, Inflammation, PDS, Saliva, Stress

## Abstract

**Background:**

Postpartum dysgalactia syndrome (PDS) is associated with a significantly higher activation of the inflammatory and stress response at parturition than in the healthy sow. Therefore, reliable and possibly non-invasive biomarkers for substantial increases of inflammation are searched to support the PDS diagnosis. This report studies the possible changes of the inflammatory marker enzyme adenosine deaminase (ADA) in serum and saliva of 38 PDS positive sows (PDS+) and 38 healthy sows (PDS-). Sampling was performed every 24 h from 60 h before to 36 h after parturition. Isoenzyme 1 (ADA1) and isoenzyme 2 (ADA2), as well as total ADA (tADA), were measured and their statistical association with several serum and saliva biomarkers of inflammation and stress was investigated.

**Results:**

Compared to a baseline (60 to 36h *prepartum*), salivary activities of ADA1, ADA2 and tADA increased significantly over time in both PDS+ and PDS- sows, reaching their peaks after parturition. In serum from PDS- sows, no changes were observed over time in either ADA1, ADA2 or tADA. In PDS+ sows, serum ADA2 activity decreased temporarily after parturition followed by a significant increase compared to baseline. ADA1, ADA2 and tADA were all significantly associated with several inflammatory biomarkers and ADA1 in serum was associated with serum cortisol. Although serum activity was higher in PDS+ than in PDS- sows, the differences were not statistically significant. Further, no difference was noted between the groups in the analyses of saliva.

**Conclusions:**

Salivary ADA1 and ADA2 increased in all sows after parturition, potentially as a response to the postpartum inflammation. However, no difference in the activity of ADA1, ADA2 and tADA were found between PDS+ and PDS- sows indicating inability to diagnose PDS under the conditions described in this report.

**Supplementary Information:**

The online version contains supplementary material available at 10.1186/s12917-021-03067-6.

## Background

Adenosine deaminase (ADA, EC number 3.5.4.4) is an enzyme present in most organic tissues, especially those of lymphoid origin [1]. Two isoenzymes have been described, of which isoenzyme 1 (ADA1) is the predominant form in lymphoid tissues. ADA1 mediates B and T cell differentiation [2] and macrophage maturation [3, 4]. Although isoenzyme 2 (ADA2) function is poorly understood, it seems to be involved in reactions by the haematopoietic system [5, 6] and regulation of the immune system, since lack of ADA2 can lead to immunosuppression and autoimmune conditions [7, 8]. Increased ADA activity has been proposed as a biomarker of cell-mediated immunity, and its increase in serum can be considered a marker of inflammation [9]. In humans, increased ADA activity has been found in several inflammatory disorders such as tuberculosis [10], visceral leishmaniasis [11], celiac disease [12], ulcerative colitis [13], typhoid fever [14], and malignancies [15–17].

Saliva is commonly used in studies on stress in humans and animals since its collection causes a minimum of disturbance [18]. However, saliva can also be used to measure biomarkers for systemic alterations such as inflammation [19]. Increased ADA activity has been found in saliva in humans with oral malignancies [20] and Sjögren’s syndrome [21]. Salivary ADA increased in bitches with pyometra and in pigs with lameness, whereas no increase was found in sera from the same animals [22]. ADA activity was more than 100-fold higher in pig saliva than in serum. In addition, since a correlation between salivary ADA and serum ADA seems to be absent, it is possible that ADA, sampled from the oral cavity, could provide information that differ from that of ADA from serum [23]. Thus, ADA in saliva may be expected to be a useful marker of inflammation in pigs.

The postpartum dysgalactia syndrome (PDS) is a common disorder in sows, appearing within the first 72 h after parturition [24], that consists of a decrease in milk production. The pathogenesis of PDS is insufficiently elucidated, as lined out by Martineau et al. [25]. Yet, activation of the stress systems (sympathetic adrenomedullary and hypothalamic adrenocortical axes), oxidative stress, metabolic changes due to low energy intake, and inflammation have been suggested to be involved in the PDS pathogenesis [26–28]. Although some clinical situations could lead to a decreased milk production, such as mastitis or metritis [29], many cases are sub-clinical and difficult to detect and therefore only discovered once piglets start to lose weight. Thus, increased piglet mortality has been reported in litters from PDS affected sows [30, 31].

In a recent report, Contreras-Aguilar et al. [32] demonstrated increased salivary ADA activity in sows after farrowing and suggested a possible relationship between the increased activity and peripartum inflammation and tissue damage. To the authors’ knowledge, ADA has not yet been studied in sows with PDS. The objective of the present study was therefore to evaluate the relationship between ADA and inflammation in sows after farrowing, as well as its potential as a biomarker for sows suffering from PDS. For this purpose, activities of the ADA1 and ADA2 isoenzymes and of total ADA (tADA) in saliva and serum obtained throughout the periparturient period of 38 PDS affected (PDS+) and 38 healthy (PDS-) sows, were analyzed. In addition, the association between the levels of ADA and other inflammatory and stress markers was evaluated.

## Results

### Saliva

The results obtained in saliva are given in Table 1 along with all *p*-values. In the healthy sows, ADA1 and tADA increased significantly in the period D (− 12 to 0 h) with the highest values obtained in the periods E (0 to 12 h) and F (12 to 24 h), respectively. ADA2 increased significantly from baseline and peaked in period E. In the PDS+ group, ADA1, ADA2 and tADA were significantly increased in period E (0 to 12 h) as compared to the baseline values, and the highest value was obtained in period F (12 to 24 h). There were no interaction between time and groups, and likewise no significant differences in ADA activities between PDS+ and PDS- sows (*p*_ADA1_ = 0.1185, *p*_ADA2_ = 0.3541 and *p*_tADA_ = 0.0718).

**Table 1:** Mean adenosine deaminase 1 (ADA1), adenosine deaminase 2 (ADA2) and total adenosine deaminase (tADA) activities (IU/L) in saliva from 38 sows with postpartum dysgalactia syndrome (PDS+) and 38 healthy sows (PDS-) in the periparturient period. Values for period B to G are compared to baseline (period A), where 0 h is the birth time of the first piglet and significant differences are indicated by the *p* -values

Parameter	Time period	PDS+ (*n* = 38)				PDS- (*n* = 38)			
		Estimate	Confidence interval		*P*-value	Estimate	Confidence interval		*P*-value
		IU/L	Lower	Upper		IU/L	Lower	Upper	
Salivary ADA1	A. (−60 to −36 h)	504.90	295.61	862.35		423.63	248.06	723.49	
	B. (−36 to −24 h)	479.31	301.33	762.43	ns	617.09	384.23	991.08	ns
	C. (−24 to −12 h)	1127.52	593.30	2142.79	ns	547.30	266.43	1124.27	ns
	D. (−12 to 0 h)	704.24	446.64	1110.43	ns	1017.66	633.24	1635.44	< 0.05
	E. (0 to 12 h)	2493.48	1054.12	5898.23	< 0.01	1936.70	867.93	4321.58	< 0.01
	F. (12 to 24 h)	4679.74	2741.66	7987.84	< 0.0001	1820.54	1095.38	3025.79	< 0.001
	G. (24 to 36 h)	3826.27	1036.87	14,119.82	< 0.01	1615.90	520.80	5013.75	< 0.05
Salivary ADA2	A. (−60 to −36 h)	5.82	3.71	9.14		5.26	3.35	8.24	
	B. (−36 to −24 h)	6.62	4.43	9.88	ns	7.08	4.75	10.57	ns
	C. (− 24 to −12 h)	12.40	7.19	21.39	ns	9.56	5.20	17.57	ns
	D. (−12 to 0 h)	9.75	6.63	14.35	ns	8.53	5.71	12.75	ns
	E. (0 to 12 h)	29.30	14.23	60.32	< 0.001	36.39	18.52	71.51	< 0.0001
	F. (12 to 24 h)	53.15	33.90	83.35	< 0.0001	21.80	14.20	33.47	< 0.0001
	G. (24 to 36 h)	38.14	12.83	113.41	< 0.01	33.58	13.02	86.61	< 0.001
Salivary tADA	A. (−60 to −36 h)	530.17	319.18	880.61		450.74	271.62	748.01	
	B. (− 36 to −24 h)	495.49	315.34	778.55	ns	626.63	398.88	984.43	ns
	C. (−24 to −12 h)	1153.49	617.20	2155.76	ns	569.94	282.82	1148.56	ns
	D. (−12 to 0 h)	722.82	463.94	1126.17	ns	940.76	603.86	1465.62	< 0.05
	E. (0 to 12 h)	2567.35	1111.60	5929.58	< 0.01	1698.14	808.28	3529.96	< 0.01
	F. (12 to 24 h)	4875.36	2937.35	8092.02	< 0.0001	1838.77	1121.65	3014.39	< 0.0001
	G. (24 to 36 h)	3863.69	1086.45	13,740.26	< 0.01	1623.42	540.32	4877.66	< 0.05

### Serum

The results of the serum analyses are shown in Table 2. In the PDS+ sows, serum ADA2 activity was significantly decreased in period E (0 to 12 h), followed by a significant increase in the periods F (12 to 24 h) and G (24 to 36 h). In contrast, there were no significant changes over time in serum ADA1 and tADA activity when compared to baseline A (− 60 to − 36 h). No changes were observed in either ADA1, ADA2 or tADA throughout the study in the PDS- sows (all *p*-values are given in Table 2). No interaction was found between time and groups and, despite a strong trend for ADA2 (*p* = 0.0514), no significant difference was seen between the PDS+ and the PDS- sows (*p*_ADA1_ = 0.0715 and *p*_tADA_ = 0.2417).

**Table 2:** Mean adenosine deaminase 1 (ADA1), adenosine deaminase 2 (ADA2) and total adenosine deaminase (tADA) activities (IU/ml) in serum from 38 sows with postpartum dysgalactia syndrome (PDS+) and 38 healthy sows (PDS-) in the periparturient period. Values for period B to G are compared to baseline (period A), where 0 h is the time of birth of the first piglet

Parameter	Time period	PDS+ (*n* = 38)				PDS- (*n* = 38)			
		Estimate	Confidence interval		*P*-value	Estimate	Confidence interval		*P*-value
		IU/ml	Lower	Upper		IU/ml	Lower	Upper	
Serum ADA1	A. (−60 to − 36 h)	2.40	1.98	2.92		2.26	1.87	2.71	
	B. (−36 to −24 h)	2.20	1.87	2.57	ns	2.07	1.74	2.45	ns
	C. (−24 to −12 h)	2.54	1.90	3.38	ns	2.33	1.76	3.10	ns
	D. (−12 to 0 h)	2.15	1.83	2.52	ns	2.50	2.12	2.94	ns
	E. (0 to 12 h)	2.49	1.87	3.32	ns	3.20	2.33	4.41	ns
	F. (12 to 24 h)	2.49	2.08	2.98	ns	2.24	1.89	2.66	ns
	G. (24 to 36 h)	2.76	1.94	3.92	ns	2.15	1.48	3.14	ns
Serum ADA2^a^	A. (−60 to −36 h)	3.28	2.89	3.71		3.06	2.72	3.46	
	B. (−36 to −24 h)	3.38	3.04	3.75	ns	3.02	2.70	3.37	ns
	C. (−24 to −12 h)	3.52	2.93	4.22	ns	3.06	2.56	3.66	ns
	D. (−12 to 0 h)	3.34	3.00	3.72	ns	3.08	2.77	3.43	ns
	E. (0 to 12 h)	2.79	2.32	3.34	< 0.0001	2.86	2.35	3.49	ns
	F. (12 to 24 h)	3.68	3.27	4.14	< 0.0001	2.81	2.51	3.14	ns
	G. (24 to 36 h)	3.40	2.73	4.25	< 0.001	3.08	2.44	3.89	ns
Serum tADA	A. (−60 to −36 h)	5.83	5.20	6.54		5.47	4.90	6.11	
	B. (−36 to −24 h)	5.68	5.16	6.25	ns	5.25	4.74	5.81	ns
	C. (−24 to −12 h)	6.14	5.19	7.27	ns	5.38	4.56	6.35	ns
	D. (−12 to 0 h)	5.58	5.07	6.15	ns	5.65	5.11	6.24	ns
	E. (0 to 12 h)	5.59	4.73	6.62	ns	6.15	5.12	7.38	ns
	F. (12 to 24 h)	6.27	5.62	6.98	ns	5.24	4.73	5.80	ns
	G. (24 to 36 h)	6.01	4.90	7.38	ns	5.57	4.49	6.92	ns

^a^ No significant interaction between *time* and *case-control* was found for ADA2 in serum. However, an overall strong trend for differences between PDS+ and PDS- (*p* = 0.0514) was estimated using the statistic model B (OUTCOME PARAMETERij = μ + TIMEi + GROUPj + ε)

### Associations between ADA1, ADA2, tADA and concentrations of inflammatory markers in saliva and serum

Salivary ADA1, ADA2 and tADA were negatively associated with white blood cell counts (WBC) and with neutrophil and lymphocyte counts in plasma, and ADA1 and tADA were also negatively associated with iron (Fe) in serum. Salivary ADA1, ADA2 and tADA were positively associated with tumour necrosis factor α (TNF-α), interleukin 6 (IL-6), serum amyloid A (SAA), C-reactive protein (CRP), haptoglobin (Hp), albumin (ALB), serum cortisol, salivary cortisol, chromogranin A (CgA) and rectal temperature. No associations were found between any of the isotypes of ADA and 8-epi prostaglandin F2 Alpha (8-epi-PGF2 α) or heart rate (Table 3; *p*-values are given in the table).

**Table 3:** Association between adenosine deaminase 1 (ADA1), adenosine deaminase 2 (ADA2), total adenosine deaminase (tADA) as analysed in serum, and inflammatory, hormonal and clinical markers in saliva*, serum** and plasma*** from 76 sows in the periparturient period. Positive associations are indicated by (+) in front of the value analysed, and negative association are indicated by (−) symbols

	Saliva						Serum					
	ADA1		ADA2		tADA		ADA1		ADA2		tADA	
	F	*P*-value	F	*P*-value	F	*P*-value	F	*P*-value	F	*P*-value	F	*P*-value
White blood cells (WBC)***	−19.43	< 0.0001	−8.64	0.01	−18.91	< 0.0001	−2.97	ns	−15.49	0.0001	−10.83	< 0.01
Neutrophils***	−10.69	< 0.01	−6.62	< 0.05	−10.03	< 0.01	−0.62	ns	−7.58	< 0.01	−3.86	0.05
Lymphocytes***	−30.66	< 0.0001	−14.56	< 0.001	−32.28	< 0.0001	− 3.54	ns	−14.98	< 0.001	− 11.33	< 0.001
Tumor necrosis factor-α (TNF-α)**	+ 59.18	< 0.0001	+ 42.06	< 0.0001	+ 61.98	< 0.0001	+ 2.15	ns	+ 22.34	< 0.0001	+ 12.21	< 0.001
Interleukin 6 (IL-6)**	+ 33.41	< 0.0001	+ 19.80	< 0.0001	+ 34.21	< 0.0001	+ 15.11	ns	+ 12.49	< 0.05	+ 25.26	ns
Serum amyloid A (SAA)**	+ 42.75	< 0.0001	+ 22.85	< 0.0001	+ 45.06	< 0.0001	+ 0.19	ns	+ 10.53	< 0.01	+ 3.80	< 0.05
C-reactive protein (CRP)**	+ 42.50	< 0.0001	+ 31.86	< 0.0001	+ 44.27	< 0.0001	+ 0.70	ns	+ 6.10	< 0.05	+ 3.62	ns
Haptoglobin (Hp)**	+ 29.53	< 0.0001	+ 30.90	< 0.0001	+ 31.94	< 0.0001	−3.36	ns	+ 12.29	< 0.001	+ 0.01	ns
Iron (Fe)**	−7.39	< 0.01	−1.90	ns	−7.90	< 0.01	−0.34	ns	−3.16	ns	−2.06	ns
Albumin (ALB)**	+ 5.84	< 0.05	+ 5.44	< 0.05	+ 5.03	< 0.05	+ 0.51	ns	+ 2.31	ns	+ 1.95	ns
8-epi prostaglandin F2α (8-epi-PGF2 α)**	−2.82	ns	−0.10	ns	−2.80	ns	−0.76	ns	+ 0.89	ns	−0.06	ns
Cortisol**	+ 25.17	< 0.0001	+ 18.71	< 0.0001	+ 24.43	< 0.0001	+ 7.88	< 0.01	+ 9.82	< 0.01	+ 14.74	< 0.001
Cortisol *	+ 55.21	< 0.0001	+ 45.37	< 0.0001	+ 56.39	< 0.0001	+ 1.88	ns	+ 1.53	ns	+ 2.69	ns
Chromogranin A (CgA)*	+ 9.36	< 0.01	+ 13.91	< 0.001	+ 9.54	< 0.01	−0.41	ns	−0.82	ns	−0.88	ns
Rectal temperature (°C)	+ 69.28	< 0.0001	+ 65.92	< 0.0001	+ 71.16	< 0.0001	+ 1.34	ns	+ 13.62	< 0.001	+ 7.60	< 0.01
Heart rate (beats per min)	+ 0.80	ns	−0.56	ns	+ 0.76	ns	− 0.43	ns	− 0.17	ns	+ 0.54	ns

In serum, ADA1 activity was positively associated with serum cortisol. ADA2 were negatively associated with WBC, neutrophil and lymphocyte counts, and positively associated with TNF-α, IL-6, SAA, CRP, Hp, serum cortisol and rectal temperature. Activity of tADA was negatively associated with WBC, neutrophil and lymphocyte counts, and positively associated with TNF-α, SAA, serum cortisol and rectal temperature (Table 3; *p*-values are given in the table).

## Discussion

The occurrence inflammatory response during the parturition process has previously been described for sows [33, 34], mares [35], cows [36] and humans [37]. In sows, this inflammation is activated from 12 h *antepartum* and peaked 12 to 36 h *postpartum*, probably due to cytokine release as a consequence of tissue trauma to the birth canal [26]. In addition, cytokines, chemokines and immunomodulatory proteins are synthesized in the placenta and gestational membranes. The patterns of expression of those cytokines suggest that inflammatory activation occurs modestly with term labor, but much more when disturbances such as preterm delivery or intrauterine infection occur [38]. Studying such inflammation in healthy sows is important in order to detect inflammation due to periparturient diseases, and biomarkers could be of great benefit for this purpose, as it has been described for WBC, neutrophil and lymphocyte counts, and concentrations of TNF-α, IL-6, Hp and Fe, for detecting PDS+ sows [26]. The possible association of serum and salivary ADA with periparturient inflammation, as well as its usefulness for detecting periparturient diseases such as PDS, has still not been studied.

Due to the significant changes observed in ADA1, ADA2 and tADA activities throughout the periparturient period in both the PDS+ and the PDS- sows, salivary ADA seems to reflect inflammatory response at peripartum, as previously described [32]. Although values of salivary ADA were higher in the PDS+ than in the PDS- sows, the differences were not statistically significant. This may be due to the high inter-individual variability observed in the animals. Therefore, ADA in our clinical setting was of lower value in the differentiation between PDS+ and PDS- sows as compared to other inflammatory markers, that previously demonstrated their ability to differentiate between these sows [26, 28]. Ideally, ADA should have been measured in a period beyond 36 h post-partum, especially since salivary ADA was still elevated after that time. Unfortunately, this was not possible as PDS sows were diagnosed as early as 6 to 6–26.9 h after birth of the first piglet and had to be excluded from the study at this time due to medical treatment.

Although serum ADA2 showed a strong trend to differ between PDS+ and PDS- sows, statistically this marker appears unsuitable for the use in the diagnosis of PDS. The relation between serum ADA and inflammation is, however, not entirely clear, as although Fávero et al. [36] demonstrated increased ADA activities in cows at the end of pregnancy and after calving, other reports were not able to demonstrate any increase in serum ADA during inflammatory conditions [22, 23]. Increased serum ADA activity at the end of gestation and in the periparturient period may be explained by two mechanisms. One is the synthesis by the placenta, since ADA is produced in the placenta and serum levels have shown to increase during pregnancy in humans [39, 40]. Another explanation could be inflammation-related synthesis of ADA, as suggested by our results.

The significant associations between salivary ADA1, ADA2 and other inflammatory markers corroborate that these isoenzymes are indeed markers of inflammation in sows. This is in agreement with previous studies showing an increased salivary ADA activity in pigs undergoing inflammatory processes [22, 23]. The role of the adenosine pathway in the inflammatory process has been fully explored in humans in several diseases such as asthma, arthritis, sepsis [41], colitis [42] or spinal cord injury [43]. TNF-a and other pro-inflammatory cytokines increase the adenosine A_2A_ receptor which activation downregulates inflammation [44] reducing pro-inflammatory cytokines and neutrophil functions [45, 46]. In contrast, serum ADA1, ADA2 and tADA appears to be less suitable markers of inflammation in sows.

## Conclusions

Salivary activity of ADA1 and ADA2 increased in sows after parturition and the activities were associated with the concentrations of several inflammatory markers. This supports the role of ADA as a marker of inflammation, but activities of ADA1, ADA2 and tADA did not significantly differ between PDS+ sows and PDS- sows. Further investigations are needed on the cause of the increased enzyme activity during the periparturient period, on the significance of ADA as an inflammatory marker, and its excretion from placenta.

## Methods

### Experimental design

This study was performed by using saliva and sera samples from a previous study in which sows were sampled in a Danish Specific Pathogen-Free sow farm [26, 27, 47]. The sows were of the Danish cross-breed (Landrace/Yorkshire). From 1 week before parturition until 3 weeks after parturition, the sows were housed in confined crates with partly slatted floors (2/3 solid concrete and 1/3 iron bars) measuring 1.6 × 2.6 m^2^. Liquid feed was fed 4 times a day and straw was assigned according to the Danish law of animal welfare. All sows were sampled every 24 h from 60 h before parturition was expected and until PDS occurred. This happened to occur within 36 h after parturition of the first piglet, and for ethical reasons, those PDS+ sows were medically treated with systemic antibiotics and anti-inflammatories and withdrawn from the study. Sows that farrowed prematurely or were treated for other reasons than PDS were also excluded.

Before each morning feeding, saliva was sampled by additive-free cotton swabs (Salivette® Cortisol, Hounissen, Denmark), immediately centrifuged for 5 min at 1000×g, and stored at -80 °C until analysis for CgA and cortisol as previously described [26]. After the morning feeding, each sow underwent a thorough clinical examination that included measurements of the body temperature and the heart rate. Thereafter, blood was sampled from *v. jugularis* using tubes with the addition of EDTA (BD, New Jersey, US) for the analyses of total WBC, lymphocyte and neutrophil counts, and additive-free tubes (BD, New Jersey, US) were centrifuged at 3000×g for 10 min. and used in the analyses of serum cortisol, Fe, ALB, IL-1, IL-6, TNF-α, SAA, Hp, CRP, and 8-epi-PGF2α, as previously described [26]. In the statistical analyses, the samples were retrospectively divided into seven different periods in relation to parturition of the first piglet (0 h): A. (− 60 to − 36 h); B. (− 36 to − 24 h); C. (− 24 to − 12 h); D. (− 12 to 0 h); E. (0 to 12 h); F. (12 to 24 h); and G. (24 to 36 h p.p.). Based on the clinical examinations, the sow was diagnosed with PDS (PDS+) if she fulfilled two out of three criteria: 1) reduced appetite (the trough were not emptied within 30 min after feeding); 2) inflammation of the udder (redness, swelling and increased skin temperature of the udder); 3) rectal temperature ≥ 39.5 °C. Based on the same criteria, the PDS+ sows were retrospectively matched with sows that remained healthy (PDS-) according to Kaiser et al. [26]. Following this criteria, 38 sows were classified as PDS+, and other 38 sows were selected as PDS- for the study.

### ADA analyses

Saliva and serum were analysed for ADA1, ADA2 and tADA activities using a commercially available, spectrophotometric, automated assay (Adenosine Deaminase assay kit, Diazyme Laboratories, Poway, CA), based on the method described by Galanti et al. [48]. In the analyses of ADA2 and tADA, the measurements were performed in the presence and absence, respectively, of erythro-9-(2-hydroxy-3-nonyl) adenine (EHNA), a specific ADA1 inhibitor [49] and ADA1 was calculated as the difference between the two measurements. The method was adapted to an automated analyzer (Olympus AU400, Olympus Diagnostica GmbH, Ennis, Ireland) following the manufacturer’s instruction with some modifications for its use in porcine saliva [22].

### Statistical analyses

Two autoregressive linear regression models (A and B) were used in the statistical analysis by the PROC MIXED procedure of Statistic Analytical Software, Enterprise Guide 7.1 (SAS® Institute, Cary, North Carolina, USA). Model A: OUTCOME PARAMETER_ij_ = μ + TIME_i_ + GROUP_j_ + TIME*GROUP_ij_ + ε_ij,_ where OUTCOME PARAMETER_ij_ indicates ADA1, ADA2, tADA values for salivary and serum; μ was the value of the observations at time 0; TIME_i_ was the explanatory variable time intervals (A to G); GROUP_j_ was the explanatory variable “PDS+/PDS-”; TIME*GROUP_ij_ was the interaction between “PDS+/PDS-” and time intervals (A to G), and ε_ij_ was the random residual error term. If significant interaction occurred by the use of model A, differences between PDS+ and PDS- and differences between the seven different time intervals were accepted. In case of non-significant interaction, model B was used instead of model A: OUTCOME PARAMETER_ij_ = μ + TIME_i_ + GROUP_j_ + ε_ij_. The effect was considered non-significant if changes in TIME_i_ were non-significant. The significance level was *p* <  0.05. The sows body condition score and parity were incorporated as explanatory variables. To improve normality of residuals plots, natural logarithm transformation was used in the analysis of ADA1 (salivary and serum), ADA2 (salivary) and tADA (salivary and serum). The association between the values of ADA1, ADA2, tADA (salivary and serum) and WBC, neutrophils, lymphocytes, TNF-α, IL-6, SAA, CRP, Hp, Fe, ALB, 8-epi-PGF2α, cortisol (salivary and serum), CgA, rectal temperature and heart rate was tested by a regression analysis in the PROC MIXED procedure of Statistic Analytical Software, Enterprise Guide 7.1 (SAS® Institute, Cary, North Carolina, USA).

## Supplementary Information

**Additional file 1.**

**Additional file 2.**

**Additional file 3.**

## Data Availability

The datasets used and analysed during the current study is available from the corresponding author on reasonable request.

## Abbreviations

ADA: Adenosine deaminase (ADA1 for isoenzyme 1, ADA2 for isoenzyme 2, and tADA for total activity)
ALB: Albumin
CgA: Chromogranin A
CRP: C-reactive protein
EHNA: Erythro-9-(2-hydroxy-3-nonyl) adenine
8-epi-PGF2α: 8-epi prostaglandin F2 Alpha
Fe: Iron
Hp: Haptoglobin
IL-6: Interleukin 6
LSMEANS: Least-squares means
PDS: Post-partum dysgalactia syndrome (PDS+ is cases and PDS- is healthy sows)
SD: Standard deviation
TNF-α: Tumor necrosis factor α
WBC: White blood cell
