# Supplementary material for: Changes of adenosine deaminase activity in serum and saliva around parturition in sows with and without postpartum dysgalactia syndrome
Source: BMC Vet Res. 2021 Nov 18;17:352. doi: 10.1186/s12917-021-03067-6 (PMC8600890; doi:10.1186/s12917-021-03067-6)
Supplement: Supplementary file 1 — Additional file 1. [file 12917_2021_3067_MOESM1_ESM.pdf]

KONTRAKT
DEN RULLENDE AFPRØVNING

Videncenter for Svineproduktion

**MELLEM**	**Jørn Skov Jensen**<br>**Møgelbjergvej 8**<br>**8762 Flemming**<br><br>**Tlf. 4074 3364**	**CHR nr.: 24050**<br><br>**E-mail: rosen-lund@os.dk**<br>**SE nr. / CVR:**
**OG**	Videncenter for Svineproduktion, Landbrug & Fødevarer<br>*VFU, Marianne Kaiser*<br>Axeltorv 3<br>1609 København V<br>Tlf. 3339 4000	
**OM**	Afprøvning nr.: 1278 Den højtydende so	

****

1. FORMÅL

1.1.
- At udføre et forstudie og blive klogere på, hvordan søer med PDS "ser ud" og hermed redegøre for søers sygdomsbillede i forbindelse med faring
- At lede efter tidlige tegn på PDS (afvigelser i blodprøvetest, spyttest, mælkeprøver, adfærd og tegn på sygdom) til brug i anden VSP-aktivitet

2. OMFANG OG GENNEMFØRELSE

2.1. Afprøvningen omfatter soholdet.

2.2. Arbejdsplanen for afprøvningen angiver, hvorledes afprøvningen skal gennemføres med hensyn til opdeling af dyrene i grupper, og hvilke registreringer, undersøgelser og analyser der skal foretages (bilag).

2.3. De dyregrupper der indgår i forsøg skal opstaldes efter gældende regler, og syge dyr skal håndteres på en forsvarlig måde (se bilag).

2.4. Den rullende Afprøvning forbeholder sig ret til at bestemme, hvilke produkter der skal afprøves og hvor de skal købes.

2.5. Supplerende registreringer vedrørende f.eks. staldklima og sygdomme skal efter nærmere aftale kunne gennemføres af de institutioner, der samarbejder med Den rullende Afprøvning.

3. KRAV TIL KONTROL OG REGISTRERING

3.1. Forudsætningerne for kontrol med foder og produktion, derunder sotavler m.v., skal være opfyldt af besætningsejeren.

3.2. Alle produktionstekniske oplysninger skal være tilgængelige, herunder bilag på køb og salg af svin samt bilag vedrørende foder og medicinindkøb.

3.3. Flytning af dyr fra en afprøvningsgruppe til en anden må kun ske efter de retningslinier, der er angivet af Den rullende Afprøvning.

3.4. Bygnings- og/eller produktionsmæssige ændringer i afprøvningsperioden skal aftales med Den

rullende Afprøvning.

3.5. Afprøvningens resultater må ikke påvirkes til egen eller andres fordel.

3.6. I kontraktperioden må der i besætningen ikke udføres afprøvninger for andre parter end Den rullende Afprøvning.

3.7. Besætningsejeren er indforstået med, at besætningens praktiserende dyrlæge og produktionsrådgiver orienteres om afprøvningens forløb, herunder modtager relevante analyseresultater, foreløbige resultater fra afprøvningen, besøgsrapporter og at besætningens rådgivere kan deltage i besætningsbesøg på foranledning af den projektansvarlige medarbejder fra Videncenter for Svineproduktion, Landbrug & Fødevarer, som betaler eventuelt honorar til de deltagende.

3.8. Besætningsejeren er indforstået med, at landbrugsteknikeren fra Den rullende Afprøvning sender E-kontrol data til det lokale rådgivningskontor.

4. BESÆTNINGSBESØG / ORIENTERING / ANALYSER

4.1. Efter behov foretager en landbrugstekniker fra Den rullende Afprøvning kontrolbesøg i besætningen. Landbrugsteknikeren foretager indsamling af data, samt medvirker ved mærkning, gruppeopdeling og vejning af grise, i den udstrækning det vedrører afprøvningen, med mindre det aftales, at besætningsejeren selv foretager dette arbejde. I øvrigt henvises der til den vedlagte arbejdsplan for projektet.

4.2. Under fortrolighed orienteres om resultaterne af de undersøgelser, der foretages i besætningen.

4.3. Der foretages analyser af bl.a. foder i den udstrækning det anses for nødvendigt.

5. OPHØR

5.1. Kontrakten er gældende indtil 1/12 2014

5.2. Kontrakten er indtil 1/12 2014 uopsigelig fra både besætningsejerens og Den rullende Afprøvnings side, jf. dog punkt 5.3.

5.3. Hvis der under afprøvningen opstår væsentlige uforudsete produktionsmæssige ulemper tages planen for afprøvningen op til fornyet overvejelse. Kan der ikke findes en løsning, kan begge parter opsige aftalen om den igangværende afprøvning med 1 måneds varsel.

5.4. Hvis kontrakten misligholdes af besætningsejeren, kan Den rullende Afprøvning ophæve kontrakten uden varsel.
Besætningsejeren skal straks tilbagebetale den kompensation, jf. punkt 7., der måtte være udbetalt i forbindelse med afprøvningens gennemførelse, samt udlevere de fast- og løstmonterede dele, der tilhører afdelingen, jf. punkt 9.2.

6. BESØGSREGLER

6.1. Besætningsejeren har ved kontraktens indgåelse oplyst, at besætningens sundhedsstatus er SPF + myc. I tilfælde af salgsstop eller sygdomsudbrud, hvor der er risiko for besætningens sundhedsstatus, skal Den rullende Afprøvning straks orienteres.

6.2. Med henblik på at udbrede kendskabet til de faktorer, der indgår i afprøvningsopgaven, forpligtes besætningsejeren til at modtage besøg i begrænset omfang, efter henvisning fra Den rullende Afprøvning.

6.3. Ved besøg i besætningen er ejeren indforstået med, at Videncenter for Svineproduktions generelle besøgsregler overholdes. Det vil sige en karantænetid på minimum 12 timer efter kontakt med svin med lavere status end ejerens besætning. Besøg fra besætning med højere sundhedsstatus eller samme sundhedsstatus, accepteres uden karantæne.

Hvis besætningsejeren har egne regler vedrørende besøg, skal disse selvfølgelig respekteres.

7. KOMPENSATION

7.1. *Der udbetales kompensation for ekstra arbejde i afprøvningsperioden. Ekstra arbejde honoreres for tiden med kr. 250,00 pr. time.*

VSP betaler alle omkostninger (materialer og løn), som er forbundet med forsøgsgrupperne. Kompensation kan gives ved: Tidsforbrug ved møder, hjælp til fiksering af søer og deslige arbejde hvor man "går til hånde". Timerne og dato anføres løbende i en udleveret notesbog.

Der kan opstå overdødelighed i de låste kuld. Dødeligheden i låste kuld sammenlignes med dødeligheden i kuld der ikke indgår i undersøgelsen. Eventuel overdødelighed kompenseres med 7 kg prisen for en gris i den aktuelle forsøgsperiode.

Der udbetales kompensation for ekstra strømforbrug ved anvendelse af diverse måleudstyr (dokumenteret via energimålere på farestaldssektion.)

Kompensation og arbejdstimer udbetales først ved afprøvningens afslutning.

Til udbetaling af kompensation fremsendes en regning til
L&F, VSP
Att. Marianne Kaiser
Projektnr. 7140400750
Vinkelvej 11
8620 Kjellerup

8. ANSVAR

8.1. I henhold til det i punkt 7.1. nævnte.

8.2. Den rullende Afprøvnings erstatningsansvar kan ikke overstige det ovenfor fastsatte.

9. OMBYGNINGSUDGIFTER

9.1. Udgifter i forbindelse med ændring eller installation af fodringsanlæg, opsætning af siloer og vægte, indkøb af fodervogne m.m., som kan henføres til en given afprøvningsopgave, dækkes efter aftale med Den rullende Afprøvning.

9.2. Vægte, fodervogne, fodringsanlæg, siloer og andet fast anlæg og inventar, der er indkøbt på Den rullende Afprøvnings regning, er dennes ejendom, hvis ikke andet er aftalt.

9.3. Efter kontraktperiodens ophør overdrages de fastmonterede dele, f.eks. fodringsanlæg, vederlagsfrit til besætningsejeren. De løse/løstmonterede dele, f.eks. blandetank og siloer, forbeholder Den rullende Afprøvning sig ret til frit at disponere over efter kontraktens udløb. Besætningsejeren kan efter aftale overtage omtalte løse/løst monterede dele til en af afdelingen fastsat pris.

9.4. Hvis besætningsejeren jf. punkt 5.3. opsiger nærværende kontrakt i afprøvningsperioden, forbeholder Den rullende Afprøvning sig ret til frit at disponere over både de fast- og løst- monterede dele. Besætningsejeren kan dog efter aftale med Den rullende Afprøvning overtage hele anlægget til en fastsat pris.

9.5. Hvis Den rullende Afprøvning opsiger nærværende kontrakt i afprøvningsperioden jf. punkt 5.3. forholdes der med det fastmonterede anlæg og de løstmonterede dele som bestemt i punkt 9.3.

9.6. Hvis besætningsejeren går konkurs eller ejendommen går på tvangsauktion er Den rullende Afprøvning berettiget til at afhente fodringsanlæg, siloer og andet fast/løst anlæg og inventar, der er indkøbt på Den rullende Afprøvnings regning.

9.7. Det påhviler besætningsejeren at sørge for vedligeholdelse samt afholdelse af udgiften til brandsikring af de installationer, der er foretaget i forbindelse med etablering af afprøvningen.

9.8 Den rullende Afprøvning hæfter for forsøgsfaciliteter, som er etableret på ejendommen i forbindelse med afprøvningen, efter dansk rets almindelige regler. Den rullende Afprøvning hæfter derfor ikke for eventuelt driftstab, og kan ikke gøres ansvarlig for indirekte tab, som kan være opstået som følge af de etablerede forsøgsfaciliteter.

Rosenlund den 6/5

[signature]

Besætningsejer

den 26/5-14

[signature]

Afdelingschef Poul Bækbo

[signature]

Afdelingschef Finn K. Udesen
Videncenter for Svineproduktion, Landbrug & Fødevarer

Videncenter for Svineproduktion

VEJLEDENDE RETNINGSLINIER FOR OPSTALDNING AF GRISE, SAMT HÅNDTERING AF SYGE DYR, I BESÆTNINGER DER ER MED UNDER DEN RULLENDE AFPRØVNING

I afprøvninger under Den rullende Afprøvning er det en forudsætning at de dyregrupper, der indgår i afprøvningerne, opstaldes og fodres efter gældende dansk lovgivning, normer og regler, med mindre afprøvningen omfatter en test af afvigende opstaldningsforhold eller fodernormer.

Opstaldning af svin reguleres gennem en række love og bekendtgørelser som bl.a. findes på Videncenter for Svineproduktions hjemmeside http://www.vsp.lf.dk under fanebladet "Viden".

Endvidere henvises til nedenstående link til Fødevarestyrelsen
http://www.foedevarestyrelsen.dk/kontrol/Egenkontrol_med_dyrevelfaerd/Sider/Forside.aspx

Videncenter for Svineproduktion forudsætter at stærkt tilskadekomne dyr, eller alvorligt syge dyr, behandles forsvarligt. Til orientering er der vist eksempler på hvad der anses for nødvendige handlinger ved håndtering af meget syge dyr i nedenstående skema. Hvis det skønnes at dyrene ikke behandles forsvarligt, vil det ved besætningsbesøg blive påtalt overfor besætningsejeren. Hvis besætningsejeren ikke får forholdene bragt i orden efter gentagne påtaler, anføres det i afprøvningens Logbog, hvilke forhold der er påtalt. Det kan herefter komme på tale at orientere besætningens dyrlæge.

Eksempler på hændelser der kræver behandling eller aflivning af dyr

**Hændelse – søer**	**Handling**
Begyndende tegn på skuldersår	Behandling og forebyggelse. Flyttes eventuelt til sygesti.
Lam i bagpart	Aflivning
Halthed	Sygesti og behandling. Uden bedring efter 5-7 dage, aflivning.
Benbrud	Omgående nødslagtning eller aflivning
Prolaps af bør	Behandling eller aflivning
Prolaps af skede eller rectalprolaps	Behandling eller nødslagtning/aflivning

**Hændelse – slagtesvin/smågrise**	**Handling**
Lam i bagpart eller halthed	Sygesti og behandling. Uden bedring efter 5-7 dage, aflivning.
Benbrud	Nødslagtning/aflivning
