# Supplementary material for: Changes of adenosine deaminase activity in serum and saliva around parturition in sows with and without postpartum dysgalactia syndrome
Source: BMC Vet Res. 2021 Nov 18;17:352. doi: 10.1186/s12917-021-03067-6 (PMC8600890; doi:10.1186/s12917-021-03067-6)
Supplement: Supplementary file 2 — Additional file 2. [file 12917_2021_3067_MOESM2_ESM.docx]

The experimental protocol was approved in advance by the Danish Animal Experiments Inspectorate. The permit number is 2013−15−2934−00970.

The homepage of the Danish Animal Experiments Inspectorate is:

https://www.foedevarestyrelsen.dk/english/Animal/AnimalWelfare/The-Animal-Experiments-Inspectorate/Pages/default.aspx

Secretariat and Council members

Secretariat:

Head of Secretariat Tom Bengtsen.

Scientific coordinator and special consultant Leif Røge Lund.

Veterinarian Katrine Svendsen

Special consultant Louise Holm Parby.

Office Clerk Betina Scheef.

Student Charlotte Nymann Eriksen

Student Natalie Ostenfeldt Jespersen

The Council members

The Council consists of eleven experts from relevant subject areas. The council members are appointed by the Minister of Environment and Food. The head of the council is required to be a judge. In addition, one council member is appointed after consulting the Danish Council for Independent Research for Medical Sciences, one member is appointed after consulting the Danish Council for Independent Research for Technology and Production Sciences, one member is appointed after consulting the Danish Health Authority, one member is appointed after consulting the Confederation of Danish Industry, one member is appointed after consulting the disease fighting NGOs, one member is appointed after consulting The Danish Animal Ethics Council and four members are appointed after consulting the Animal Welfare Organizations. The council members are appointed for four years.

Chairman: Judge Christian Lundblad

The court in Aalborg

(appointed from 1 July 2018)

Other members:

Professor emeritus, MD, DMSc. Peter Bie,

University of Southern Denmark, Department of Molecular Medicine. Appointed by The Danish Council for Independent Research - Medical Sciences the 15th of October 2014.

Substitute: Professor Preben Dybdal Thomsen, University of Copenhagen - Department of Veterinary and Animal Sciences, Anatomy and Biochemistry

Associate professor Dorte Bratbo Sørensen,

University of Copenhagen, Department of Veterinary and Animal Sciences - Experimental Animal Models. Appointed by The Danish Council for Independent Research - Technology and Production Sciences 18th of December, 2017.

Substitute: CEO Kirstine Øvlisen, 3 R Strategy & Veterinary Services, Novo Nordic A/S

Clinical professor Lars Bo Svendsen,

Copenhagen University Hospital, Department of Clinical Medicine, Section of Surgery and Internal Medicine, Appointed by the Danish Health and Medicine Authority 15th of October 2014.

Substitute: Nellie Bering Zinther, Department of Surgery, Regional hospital Horsens

Head of Technical Operation Mikkel Lykke Jensen,

CiToxLAB Scantox A/S

Appointed by the Confederation of Danish Industry 5th of November 2017.

Substitute: Senior Vice President Helle Northeved, Non-clinical Safety Research, H. Lundbeck A/S

Professor Birgitte Holst,

University of Copenhagen, Department of Neuroscience and Pharmacology.

Appointed by the disease fighting NGOs 28th of March 2016.

Substitute: Associate Professor Kim Bak Jensen, BRIC, University of Copenhagen

Farmer Karsten Vig Jensen,

Appointed by the Board of Animal Ethics 21st of March 2015.

Substitute: Chairman Peter Mollerup, The Organisation for cooperation of animal welfare

groups (DOSO)

CEO Bente Lakjer,

The Danish Society for the Protection of Laboratory Animals

Appointed by the animal protection societies 1st of July 2018.

Substitute: Rosemary Goddard Svendsen, The Danish Society for the Protection of Laboratory Animals

Laboratory Animal Veterinarian Kirsten Rosenmay Jacobsen,

Ellegaard Gottingen Minipigs A/S. Appointed by the animal protection societies 5th of November 2017.

Substitute: Associate professor Julie Fjeldborg, University of Copenhagen, Department of Veterinary Clinical Sciences - Medicine and Surgery.

Veterinarian Louise Bundgaard

University of Copenhagen, Department of Veterinary Clinical Sciences - Medicine and Surgery. Appointed by the animal protection societies 1st of July 2018.

Substitute: Veterinarian Mogens Teken Christophersen, Department of Veterinary Clinical

Studies, University of Copenhagen (KU-SUND), Copenhagen, Denmark.

Biologist Birgith Sloth,

The Organisation for cooperation of animal welfare groups (DOSO). Appointed by the animal protection societies 15th of November 2017.

Substitute: Head of Council Mogens Wilbert, Danish Association for Cat Protection
