# Supplementary material for: Changes of adenosine deaminase activity in serum and saliva around parturition in sows with and without postpartum dysgalactia syndrome
Source: BMC Vet Res. 2021 Nov 18;17:352. doi: 10.1186/s12917-021-03067-6 (PMC8600890; doi:10.1186/s12917-021-03067-6)
Supplement: Supplementary file 3 — Additional file 3. [file 12917_2021_3067_MOESM3_ESM.pdf]

Reviewer Comments:

Reviewer 1

The work is well described ant the results support the hypothesis.

I have only a minor comment for the authors, they should be discussed about the role of adenosine pathway in the inflammatory process (like during PDS) in this context could help to cite the following article that investigated the role of adenosine pathway in several inflammatory diseases:

1) Pallio G, Bitto A, Ieni A, Irrera N, Mannino F, Pallio S, Altavilla D, Squadrito F, Scarpignato C, Minutoli L. Combined Treatment with Polynucleotides and Hyaluronic Acid Improves Tissue Repair in Experimental Colitis. Biomedicines. 2020 Oct 20;8(10):438. doi: 10.3390/biomedicines8100438.

2) Irrera N, Bitto A, Vaccaro M, Mannino F, Squadrito V, Pallio G, Arcoraci V, Minutoli L, Ieni A, Lentini M, Altavilla D, Squadrito F. PDRN, a Bioactive Natural Compound, Ameliorates Imiquimod-Induced Psoriasis through NF-κB Pathway Inhibition and Wnt/β-Catenin Signaling Modulation. Int J Mol Sci. 2020 Feb 12;21(4):1215. doi: 10.3390/ijms21041215.

3) Marini HR, Puzzolo D, Micali A, Adamo EB, Irrera N, Pisani A, Pallio G, Trichilo V, Malta C, Bitto A, Squadrito F, Altavilla D, Minutoli L. Neuroprotective Effects of Polydeoxyribonucleotide in a Murine Model of Cadmium Toxicity. Oxid Med Cell Longev. 2018 Aug 29;2018:4285694. doi: 10.1155/2018/4285694.

4) Irrera N, Arcoraci V, Mannino F, Vermiglio G, Pallio G, Minutoli L, Bagnato G, Anastasi GP, Mazzon E, Bramanti P, Squadrito F, Altavilla D, Bitto A. Activation of A2A Receptor by PDRN Reduces Neuronal Damage and Stimulates WNT/β-CATENIN Driven Neurogenesis in Spinal Cord Injury. Front Pharmacol. 2018 May 29;9:506. doi: 10.3389/fphar.2018.00506.

5) Pizzino G, Irrera N, Galfo F, Oteri G, Atteritano M, Pallio G, Mannino F, D'Amore A, Pellegrino E, Aliquò F, Anastasi GP, Cutroneo G, Squadrito F, Altavilla D, Bitto A. Adenosine Receptor Stimulation Improves Glucocorticoid-Induced Osteoporosis in a Rat Model. Front Pharmacol. 2017 Sep 5;8:558. doi: 10.3389/fphar.2017.00558.

6) Squadrito F, Micali A, Rinaldi M, Irrera N, Marini H, Puzzolo D, Pisani A, Lorenzini C, Valenti A, Laurà R, Germanà A, Bitto A, Pizzino G, Pallio G, Altavilla D, Minutoli L. Polydeoxyribonucleotide, an Adenosine-A2AReceptor Agonist, Preserves Blood Testis Barrier from Cadmium-Induced Injury. Front Pharmacol. 2017 Jan 10;7:537. doi: 10.3389/fphar.2016.00537.

7) Pallio G, Bitto A, Pizzino G, Galfo F, Irrera N, Squadrito F, Squadrito G, Pallio S, Anastasi GP, Cutroneo G, Macrì A, Altavilla D. Adenosine Receptor Stimulation by Polydeoxyribonucleotide Improves Tissue Repair and Symptomology in Experimental Colitis. Front Pharmacol. 2016 Aug 23;7:273. doi: 10.3389/fphar.2016.00273.

ANSWERS (Reviewer 1)

The authors would like to give their thanks to this reviewer for his/her help and the bibliography suggestions. After carefully read the articles, the following information has been included in the manuscript in lines 164-172: "The significant associations between salivary

ADA1, ADA2 and other inflammatory markers corroborate that these isoenzymes are indeed markers of inflammation in sows. This is in agreement with previous studies showing an increased salivary ADA activity in pigs undergoing inflammatory processes [22, 23]. The role of the adenosine pathway in the inflammatory process has been fully explored in humans in several diseases such as asthma, arthritis, sepsis [41], colitis [42] or spinal cord injury [43]. TNF-a and other pro-inflammatory cytokines increase the adenosine A_{2A} receptor which activation downregulates inflammation [44] reducing pro-inflammatory cytokines and neutrophil functions [45, 46]. In contrast, serum ADA1, ADA2 and tADA appears to be less suitable markers of inflammation in sows."

New added references:

41. Cronstein B, Pacher P, Haskó G, Linden J: Adenosine receptors: therapeutic aspects for inflammatory and immune diseases. Nature reviews Drug discovery 2008, 7(9):759-770.

42. Pallio G, Bitto A, Ieni A, Irrera N, Mannino F, Pallio S, Altavilla D, Squadrito F, Scarpignato C, Minutoli L. Combined Treatment with Polynucleotides and Hyaluronic Acid Improves Tissue Repair in Experimental Colitis. Biomedicines. 2020 Oct 20;8(10):438. doi: 10.3390/biomedicines8100438.

43. Irrera N, Arcoraci V, Mannino F, Vermiglio G, Pallio G, Minutoli L, Bagnato G, Anastasi GP, Mazzon E, Bramanti P, Squadrito F, Altavilla D, Bitto A. Activation of A2A Receptor by PDRN Reduces Neuronal Damage and Stimulates WNT/β-CATENIN Driven Neurogenesis in Spinal Cord Injury. Front Pharmacol. 2018 May 29;9:506. doi: 10.3389/fphar.2018.00506.

44. Khoa, N. D., Postow, M., Danielsson, J., and Cronstein, B. N. (2006). Tumor necrosis factor-a prevents desensitization of GaS-coupled receptors by regulating GRK2 association with the plasma membrane. Mol. Pharmacol. 69, 1311–1319. doi: 10.1124/mol.105.016857

45. Cronstein, B.N.; Rosenstein, E.D.; Kramer, S.B.; Weissmann, G.; Hirschhorn, R. Adenosine; a physiologic modulator of superoxide anion generation by human neutrophils. Adenosine acts via an A2 receptor on human neutrophils. J. Immunol. 1985, 135, 1366–1371.

46. Sullivan, G.W.; Lee, D.D.; Ross, W.G.; Di Vietro, J.A.; Lappas, C.M.; Lawrence, M.B.; Linden, J. Activation of A2A adenosine receptors inhibits expression of α4/β1 integrin (verylate antigen-4) on stimulated human neutrophils. J. Leukoc. Biol. 2004, 75, 127–134.

Reviewer 2

"Changes of adenosine deaminase activity in serum and saliva around parturition in sows with and without postpartum dysgalactia syndrome"

The study evaluated the ADA's potential as an inflammatory marker in sows suffering from postpartum dysgalactia syndrome (PDS) and also their association with serum and saliva biomarkers of inflammations. As a result, activities of ADA1, ADA2, and tADA did not significantly differ between healthy sows and those suffering PDS.

Many details and information were not included in this paper, making it difficult to understand, especially without reading some referenced papers. Therefore, I do not recommend the manuscript for publication as a full research article in Veterinary Research.

Specific comments:

Background:

- Additional information about the PDS definition is necessary to the readers better understand the context.

<u>**ANSWERS (Reviewer 2)**</u>

In lines 74-82, the information regarding PDS has been enlarged: "The postpartum dysgalactia syndrome (PDS) is a common disorder in sows, appearing within the first 72 h after parturition [24], that consist of a decrease in milk production. The pathogenesis of PDS is insufficiently elucidated, as lined out by Martineau et al. [25]. Yet, activation of the stress systems (sympathetic adrenomedullary and hypothalamic adrenocortical axes), oxidative stress, metabolic changes due to low energy intake, and inflammation have been suggested to be involved in the PDS pathogenesis [26-28]. Although some clinical situations could lead to a decreased milk production, such as mastitis or metritis [29], many cases are sub-clinical and difficult to detect, and therefore only discovered once piglets start to lose weight. Thus, increased piglet mortality has been reported in litters from PDS affected sows [30, 31]."

New added references:

29. Maes D, Papadopoulos G, Cools A, Janssens GPJ. Postpartum dysgalactia in sows: pathophysiology and risk factors. Tierärztl Prax. 2010;38(Suppl 1):15–20.

30. Quesnel H, Farmer C, Devillers N. Colostrum intake. Influence on piglet performance and factors of variation. Livest Sci. 2012;146:105–114.

31. Mirko CP, Bilkei G. Acute phase proteins, serum cortisol and prewearing litter performance in sows suffering from periparturient disease. Acta Vet (Beograd) 2004;54:153–161.

- In line 75, it is mentioned that the PDS may be present within the first 72 hours postpartum. This information creates the expectation that the analyses will be performed until this moment (72 h). However, the evaluations were performed up to 36 hours postpartum.

This reviewer is right and it was actually the original plan to study all sows for 72 hours after parturtion. However, PDS sows were excluded from the study if they were medically treated, as biological tests and clinical appearance are not valid to study after a medical treatment. Moreover, due to the Danish animal welfare act and the animal experiment permit, it was necessary to treat the sows immediately after disease was observed. And, since all PDS-sows were diagnosed and treated within the time interval of 6-26.9 hours after birth of the first piglet (reported in Kaiser et al., 2018(1)), it no longer made sense to compare the two groups in all 72 hours. Healthy control sows were actually studied for 3 days after farrowing, but these data are not included because there are no cases to compare with for the last time period. This is now elaborated in lines 187-191: "All sows were sampled every 24 h from 60 h before parturition was expected and until PDS occurred. This happened to occur within 36 h after parturition of the first piglet, and for ethical reasons, those PDS+ sows were medically treated with systemic antibiotics and anti-inflammatories and withdrawn from the study. Sows that farrowed prematurely and sows treated for reasons other than PDS were also excluded."

Although PDS was detected in all animals at 36 hours in all PDS + animals, exploring the values beyond this sampling time would have been interesting, moreover because ADA values were not normalized yet in healthy animals. This could be considered as a limitation of the study and it has been discussed in the new version of the manuscript in lines 149-152: "Ideally, ADA should have been measured in a period beyond 36 h post-partum, especially since salivary ADA was still elevated after that time. Unfortunately, this was not possible as PDS sows were diagnosed as early as 6 to 6-26.9 hours after birth of the first piglet and had to be excluded from the study at this time due to medical treatment."

Material and Methods

- Why using ÷ instead of -?

The sign ÷ has been replaced by – throughout the manuscript.

- Much important information essential to understanding the manuscript was not described, such as facilities, feeding, parity order, the health status of the sows (other problems than PDS), and other standard information regarding animals, management, and housing. Thus, detailed information about the methodology is necessary to clarify the manuscript.

As the reviewer is indicating in the next comment, the study was performed using banked samples from a previous report. This has been clarified in the text and the details this reviewer asked for are therefore indicated in that report. In addition to this, to satisfy the requirement of the reviewer, the following information has been added in lines 183-191: "The sows were of the Danish cross-breed (Landrace/Yorkshire). From 1 week before parturition until 3 weeks after parturition, the sows were housed in confined crates with partly slatted floors (2/3 solid concrete and 1/3 iron bars) measuring 1.6 × 2.6 m2. Liquid feed was fed 4 times a day and straw was assigned according to the Danish law of animal welfare. All sows were sampled every 24 h from 60 h before parturition was expected and until PDS occurred. This happened to occur within 36 h after parturition of the first piglet, and for ethical reasons, those PDS+ sows were medically treated with systemic antibiotics and anti-inflammatories and withdrawn from the study. Sows that farrowed prematurely or were treated for other reasons than PDS were also excluded."

If the reviewer thinks that more information should be provided we would be happy to do it.

- Line 168: "This study included saliva and sera from sows sampled in a Danish sow farm as previously described by Kaiser et al. [26, 27, 37]". Reading the articles from the authors, it seems to be the same animals of the present study. Thus, if they are the same, it is essential to clarify this question since ADA1 and 2 could be included in one of the previous works. Despite that, even if the articles are part of the same study, it is interesting to add the most relevant information in the present manuscript.

The reviewer is right and the study was conducted with the serum and saliva samples obtained from a previous study. According to this comment, the sentence has been modified as (lines 182-183): "This study was performed by using saliva and sera samples from a previous study in which sows were sampled in a Danish Specific Pathogen-Free sow farm [26, 27, 37]."

- Statistical analysis: autoregressive analysis was used, and comparisons were performed based on (-60 to -36 h related to the first piglet born). If PDS is a postpartum condition, why considering this interval as a baseline? The authors did not mention the interaction between

group and time. Perhaps, repeated measure analysis would fit better in this data. Additionally, authors could test the use of the first piglet born as the reference.

a) Regarding the use of considering the interval of -60 to -36 h to the first partum recorded. In previous reports in other species it has been indicated that ADA could change by the end of gestation and delivery. And in a more recent report, Contreras-Aguilar et al. (2021) demonstrated that salivary ADA increases at farrowing in sows. For this reason, the interval -60 to -36 h to the first partum recorded was used as reference because of the possibility of seeing changes in ADA levels even in healthy animals.

Authors would like to clarify this matter, and for this reason the lines 84-92 in the Background section has been modified to: “In a recent report, Contreras-Aguilar et al. [32] demonstrated increased salivary ADA activity in sows after farrowing and suggested a possible relationship between the increased activity and peripartum inflammation and tissue damage. To the authors’ knowledge, ADA has not yet been studied in sows with PDS. The objective of the present study was therefore to evaluate the relationship between ADA and inflammation in sows after farrowing, as well as its potential as a biomarker for sows suffering from PDS. For this purpose, activities of the ADA1 and ADA2 isoenzymes and of total ADA (tADA) in saliva and serum obtained throughout the periparturient period of 38 PDS affected (PDS+) and 38 healthy (PDS-) sows, were analyzed. In addition, the association between the levels of ADA and other inflammatory and stress markers was evaluated.”

Regarding the statistical approach, the statistical analysis was actually a repeated measurement-analysis. We assumed an autoregressive co-variance-structure, because it makes biological sense here, and it gave a reasonable fit for the data. Autoregressive analyses assume that there is a correlation between measurements of the dependent variable.

New added reference:

32. Contreras-Aguilar MD, López-Arjona M, Martínez-Miró S, Escribano D, Hernández-Ruipérez F, Cerón JJ, Tecles F. Changes in saliva analytes during pregnancy, farrowing and lactation in sows: A sialochemistry approach. Vet J. 2021 Jul;273:105679.

Results

- The results are confusing and, many times, are redundant. P-values are not present when authors claimed to found no evidence of an effect.

Some redundant information has been eliminated such as the sentence: “Compared to baseline A (÷ 60 to ÷ 36 h), salivary activity of ADA1, ADA2 and tADA increased significantly during the first 36 hours p.p. investigated in this study, in both the PDS + and the PDS ÷ sows.”.

In addition, p values for the non-significant comparisons have been included. In the new version of the manuscript, the results can be now read as (lines 95-102): “The results obtained in saliva are given in Table 1 along with all p-values. In the healthy sows, ADA1 and tADA increased significantly in the period D (- 12 to 0 h) with the highest values obtained in the periods E (0 to 12 h) and F (12 to 24 h), respectively. ADA2 increased significantly from baseline and peaked in period E. In the PDS + group, ADA1, ADA2 and tADA were significantly increased in period E (0 to 12 h) as compared to the baseline values, and the highest value was obtained in period F (12 to 24 h). There were no interaction between time and groups, and likewise no significant differences in ADA activities between PDS+ and PDS- sows (pADA1 = 0.1185, pADA2 = 0.3541 and ptADA = 0.0718).”

And in lines 104-111: “The results of the serum analyses are shown in Table 2. In the PDS + sows, serum ADA2 activity was significantly decreased in period E (0 to 12 h), followed by a significant increase in the periods F (12 to 24 h) and G (24 to 36 h). In contrast, there were no significant changes over time in serum ADA1 and tADA activity when compared to baseline A (÷ 60 to ÷ 36 h). No changes were observed in either ADA1, ADA2 or tADA throughout the study in the PDS ÷ sows (all p-values are given in Table 2). No interaction were found between time and groups and, despite a strong trend for ADA2 ($p = 0.0514$), no significant difference were seen between the PDS+ and the PDS- sows ($p_{ADA1} = 0.0715$ and $p_{tADA} = 0.2417$).”

Discussion

- Discussion is concise, especially about the results presented in table 3. Seeking two enzymes is little to support a full research article. Authors could explore this enzyme with more information about the parturition dynamics or the female status at the time of parturition.

According to the reviewer’s suggestion, the role of inflammation in delivery has been further discussed in lines 128-140: “The occurrence inflammatory response during the parturition process has previously been described for sows [33, 34], mares [35], cows [36] and humans [37]. In sows, this inflammation is activated from 12 h *antepartum* and peaked 12 to 36 h *postpartum*, probably due to cytokine release as a consequence of tissue trauma to the birth canal [26]. In addition, cytokines, chemokines and immunomodulatory proteins are synthesized in the placenta and gestational membranes. The patterns of expression of those cytokines suggest that inflammatory activation occurs modestly with term labor, but much more when occurring disturbances such as preterm delivery or, moreover, with intrauterine infection [38]. Studying this inflammation in healthy sows is important in order to detect inflammation due to periparturient diseases, and biomarkers could be of great benefit for this purpose, as it has been described for WBC, neutrophil and lymphocyte counts, and concentrations of TNF-α, IL-6, Hp and Fe, for detecting PDS+ sows [26]. The possible association of serum and salivary ADA with periparturient inflammation, as well as its usefulness for detecting periparturient diseases such as PDS, had not been still studied.”

New added reference:

38. Keelan JA, Blumenstein M, Helliwell RJ, Sato TA, Marvin KW, Mitchell MD. Cytokines, prostaglandins and parturition--a review. Placenta. 2003 Apr;24 Suppl A:S33-46. doi: 10.1053/plac.2002.0948. PMID: 12842412.

Tables

- Table 3 is poorly explored in the manuscript, and it seems unnecessary.

The authors think that Table 3 would be of highly interest in order to assess the possible associations between ADA and other inflammatory biomarkers, since these associations could aid in the clarification of the reason of ADA increases at farrowing.

To explore more this Table in the manuscript, the relationship between adenosine pathway and inflammation has been further discussed in the new version of the manuscript, according to the reviewer’s 1 comments. The paragraph can be read now in lines 164-172 as: “The significant associations between salivary ADA1, ADA2 and other inflammatory markers corroborate that these isoenzymes are indeed markers of inflammation in sows. This is in agreement with previous studies showing an increased salivary ADA activity in pigs undergoing

inflammatory processes [22, 23]. The role of the adenosine pathway in the inflammatory process has been fully explored in humans in several diseases such as asthma, arthritis, sepsis [41], colitis [42] or spinal cord injury [43]. TNF-a and other pro-inflammatory cytokines increase the adenosine A_{2A} receptor which activation downregulates inflammation [44] reducing pro-inflammatory cytokines and neutrophil functions [45, 46]. In contrast, serum ADA1, ADA2 and tADA appears to be less suitable markers of inflammation in sows."

- Suggestion: use graphs.

We agree with the reviewer that good illustrative graphs are always preferable. With all due respect, we would like to argue that a table is the best choice in the present case. We think the table gives a very good overview of the numerous p-values and the association to the other inflammation markers (which we believe is a rather essential result). If the results were to be given in a graph, it would require that all p-values are included in the text. This will, in our best belief, be disruptive to the reader. We therefore kindly request that the table will be kept as it is.

Reviewer 3 (Answers to this reviewer are listed after each question)

Line 27: substitute ÷ with – here and in all the text.

The sign ÷ has been replaced by – throughout the manuscript.

Line 72: substitute saliva ADA with ADA saliva.

The sentence "Thus, saliva ADA may be expected to be a useful marker of inflammation in pigs" has been modified to: "Thus, ADA in saliva may be expected to be a useful marker of inflammation in pigs"

Line 78: change the pathogenesis of PDS with PDS pathogenesis.

The sentence "…have been suggested to be involved in the pathogenesis of PDS [26–28]" has been modified to: "…have been suggested to be involved in the PDS pathogenesis [26–28]."

Line 166: please substitute Study design with Experimental design.

This change has been introduced in the text.

Please, put period at the end of every figure/table legend.

This change has been performed.
